# Cutaneous Adverse Events Following COVID-19 Vaccination in Japan: A Questionnaire Survey

**DOI:** 10.7759/cureus.80257

**Published:** 2025-03-08

**Authors:** Yuto Yamamura, Chisa Nakashima, Nana Kagawa, Yumi Aoyama, Akihisa Yamamoto, Hisao Kawahira, Yumiko Kubota, Saeko Nakajima, Takeshi Nakahara, Yoko Fuyuno, Daisuke Tsuruta, Ayaki Matsumoto, Risa Matsuo, Riichiro Abe, Akihiko Yuki, Hayato Takahashi, Chiaki Takahashi, Shin'Ichi Imafuku, Emi Sato, Susumu Fujiwara, Masahito Yasuda, Yayoi Tada, Kotaro Hayashi, Norito Katoh, Daisuke Watanabe, Atsushi Otsuka

**Affiliations:** 1 Dermatology, Kindai University, Osaka, JPN; 2 Dermatology, Kawasaki Medical School, Kurashiki, JPN; 3 Dermatology, Takarazuka City Hospital, Takarazuka, JPN; 4 Dermatology, Kagoshima University, Kagoshima, JPN; 5 Dermatology, Fukuoka Sanno Hospital, Fukuoka, JPN; 6 Dermatology, Kyoto University Graduate School of Medicine, Kyoto, JPN; 7 Dermatology, Kyushu University, Fukuoka, JPN; 8 Dermatology, Osaka Metropolitan University, Osaka, JPN; 9 Dermatology, Asahikawa Medical University, Asahikawa, JPN; 10 Dermatology, Niigata University, Faculty of Medicine, Graduate School of Medical and Dental Science, Niigata, JPN; 11 Dermatology, Niigata University, Faculty of Medicine, Graduate School of Medical and Dental Sciences, Niigata, JPN; 12 Dermatology, Keio University School of Medicine, Tokyo, JPN; 13 Dermatology, Fukuoka University, Fukuoka, JPN; 14 Dermatology, Kobe University Graduate School of Medicine, Kobe, JPN; 15 Dermatology, Gunma University Graduate School of Medicine, Gunma, JPN; 16 Dermatology, Teikyo University School of Medicine, Tokyo, JPN; 17 Dermatology, North Campus, Kyoto Prefectural University of Medicine, Kyoto, JPN; 18 Dermatology, Aichi Medical University, School of Medicine, Aichi, JPN

**Keywords:** alopecia, bullous pemphigoid, covid-19 vaccine, cutaneous adverse events, erythema multiforme, psoriasis

## Abstract

We conducted a nationwide survey in Japan to clarify the clinical spectrum of these events. An initial questionnaire was sent to 126 dermatology facilities, and responses were obtained from 66 (52.4%). Among these responding facilities, the most commonly identified cutaneous adverse events after COVID-19 vaccination were urticaria (49 (74.2%)), delayed local reactions (37 (56.1%)), erythema multiforme (31 (47.0%)), and alopecia (30 (45.5%)). In the detailed survey, the primary adverse events were EM (19 (20.9%)), bullous pemphigoid (7 (7.7%)), and alopecia (6 (6.6%)). The mean latency from vaccination to onset was 13.1 days, and the mean duration of symptoms was 74.2 days. Although this study cannot establish a direct causal relationship between vaccination and adverse events, it highlights the need for dermatologists to recognize potential cutaneous reactions and provide appropriate care.

## Introduction

The novel coronavirus disease 2019 (COVID-19), caused by severe acute respiratory syndrome coronavirus 2 (SARS-CoV-2), has spread globally since its emergence in Wuhan, China, in December 2019 [1]. The World Health Organization declared the COVID-19 outbreak a pandemic in March 2020. In Japan, the first case was confirmed in January 2020, and the cumulative number of cases surpassed 400,000 in February 2021 [2]. Following an unprecedented worldwide vaccine development race, vaccination against COVID-19 began in December 2020 in many countries [3]. In Japan, priority vaccination of healthcare workers started in February 2021, followed by vaccination of the elderly in April 2021 [4]. Mass vaccination has played a critical role in controlling the pandemic; however, adverse reactions, including dermatological side effects, have been increasingly recognized. While local reactions, such as injection site pain and swelling, are common, more diverse cutaneous adverse events have also been reported. Despite emerging global data, the clinical spectrum and characteristics of these cutaneous events remain underreported in Japan.

The majority of adverse events to COVID-19 vaccines are local events at the injection site, such as pain, swelling, and erythema, while systemic events include fever, fatigue, headache, and myalgia [5]. COVID-19 mRNA vaccination has led to reports of various cutaneous adverse events [6,7], including delayed local reactions (so-called "COVID arm" or "Moderna arm") [8,9], urticaria [10-12], vasculitis/vascular disorders [13-15], and bullous pemphigoid (BP) [16,17]. However, the clinical spectrum and characteristics of cutaneous adverse events following COVID-19 vaccination remain insufficiently described. This study aimed to clarify the impact of COVID-19 vaccination on dermatological practice by analyzing the clinical characteristics of cutaneous adverse events in Japan. Through a nationwide survey, we investigated the frequency and severity of these events, including common reaction types and disease exacerbations, to provide insights into appropriate patient management.

## Materials and methods

Study design and participants

This study is a retrospective, cross-sectional study conducted in April 2021. An initial questionnaire survey was distributed to 126 major dermatology institutions in Japan regarding cutaneous adverse events following COVID-19 vaccination (Appendix A). Responses were obtained from 66 (52.4%) out of 126 institutions. The questionnaire items included the experienced cutaneous adverse events after COVID-19 vaccination (multiple-choice).

Subsequently, we requested the institutions that responded to the initial questionnaire to participate in a detailed survey and retrospectively collected information on cases that developed cutaneous adverse events after COVID-19 vaccination (Appendix B). Inclusion criteria consisted of all cases reported in the survey. No specific exclusion criteria were applied, and all reported cases were analyzed.

Data collection and analysis

The survey items included age, sex, patient background, diagnosis of the cutaneous adverse event, treatment, latency from the last vaccination to onset, duration from onset to resolution (or remission in cases of exacerbation of skin diseases), number of vaccine doses received before the adverse event onset, presence or absence of re-vaccination after the adverse event, and vaccine brand. The vaccines administered during the study period included Pfizer-BioNTech (BNT162b2; Pfizer, New York, NY, US), Moderna (mRNA-1273; Cambridge, Massachusetts, US), and Takeda (NVX-CoV2373; Tokyo, Japan). However, this study did not perform a comparative analysis of adverse event incidence across different vaccine types due to the limited sample size and retrospective design. Descriptive analysis was performed without statistical hypothesis testing. A total of 15 (16.4%) institutions participated in the detailed survey, providing information on 91 cases of cutaneous adverse events following COVID-19 vaccination.

## Results

Initial questionnaire survey

**Figure 1:**
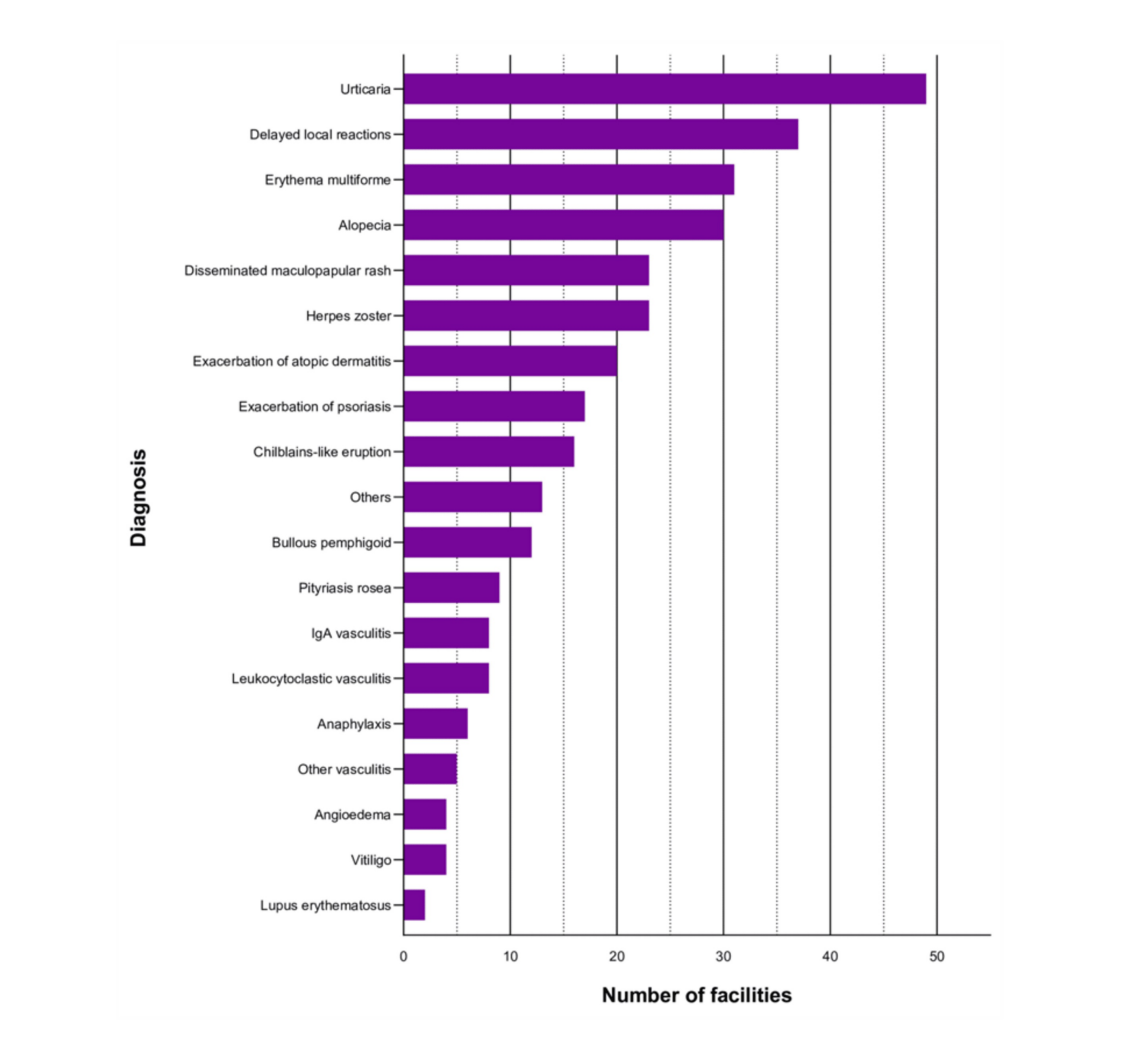
Cutaneous adverse events to COVID-19 vaccines in the initial questionnaire survey Out of the 66 facilities surveyed, the most commonly reported dermatological adverse events were urticaria (49 (74.2%)), delayed local reactions (37 (56.1%)), EM (31 (47.0%)), and alopecia (30 (45.5%)). Disseminated maculopapular rash (23 (34.8%)), herpes zoster (23 (34.8%)), exacerbation of atopic dermatitis (20 (22.0%)), and exacerbation of psoriasis (17 (25.8%)) were also frequently reported.

Responses were obtained from 66 (52.4%) out of 126 facilities. The experienced cutaneous adverse events after COVID-19 vaccination (multiple answers allowed) were urticaria (49 (74.2%)), delayed local reactions (37 (56.1%)), erythema multiforme (EM) (31 (47.0%)), and alopecia (30 (45.5%)). The cutaneous adverse events categorized as “Others” included pemphigus, butterfly rash, livedo reticularis, idiopathic thrombocytopenic purpura, acute generalized exanthematous pustulosis, exacerbation of skin induration in systemic sclerosis, Sweet's syndrome, localized scleroderma, pustular psoriasis, dermatomyositis, exacerbation of palmoplantar pustulosis, paraneoplastic pemphigus, and anhidrosis (Figure 1).

Detailed survey

Fifteen institutions participated in the detailed survey, providing information on 91 cases (40 males, 51 females) that developed cutaneous adverse events after COVID-19 vaccination. The age ranged from 18 to 95 years (mean 63.4 years). The patient background included atopic dermatitis (4 (4.4%)), psoriasis vulgaris (8 (8.8%)), a history of urticaria (1 (1.1%)), concomitant urticaria (8 (8.8%)), and concomitant malignancy (6 (6.6%)). The latency from the last vaccination to the onset of cutaneous adverse events ranged from 0 to 112 days (mean 13.1 days, median 5 days). The duration from onset to resolution (or remission in cases of exacerbated skin diseases) ranged from 0.02 to 365 days (mean 74.2 days, median 60 days), with 9 (9.9%) remaining unresolved at the time of the survey (the outcomes of 11 cases remain unknown). See Table 1.

**Table 1:** Summary of patient demographics and clinical characteristics Details of 91 cases of cutaneous adverse events following COVID-19 vaccination, including age distribution, patient background, latency to onset, and duration until resolution or remission. Outcomes of 9 (9.9%) cases were unresolved, and 11 (12.1%) cases had unknown outcomes at the time of the survey.

Characteristics		Details
Mean age		63.4 years (range: 18-95 years)
Male: female		40 (44.0%): 51 (56.0%) (cases)
Background	Atopic dermatitis	4 (4.4%) cases
	Psoriasis vulgaris	8 (8.8%) cases
	Urticaria	8 (8.8%) cases (concurrent), 1 (1.1%) case (past)
	Malignancy	6 (6.6%) cases
Latency from last vaccination to onset		13.1 days (min: Immediately, max: 112 days)
The duration from onset to resolution		74.2 days (min: 30 minutes, including ongoing cases)

**Figure 2:**
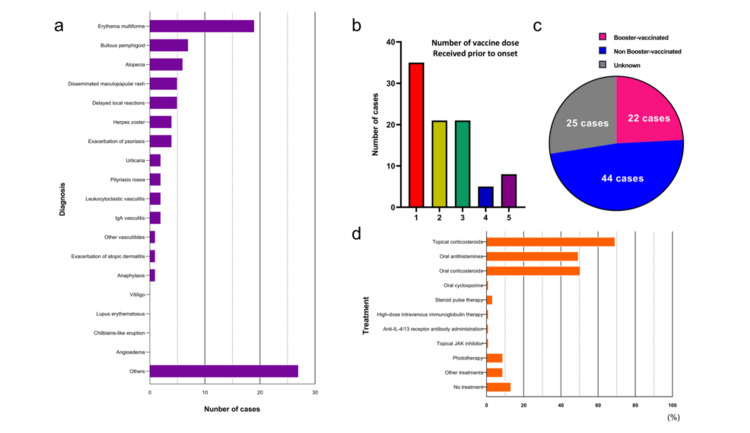
Cutaneous adverse events to COVID-19 vaccines in the detailed survey (a) A total of 91 cases of cutaneous adverse events were observed at 15 facilities in the detailed survey. The main cutaneous adverse events were EM in 19 (20.9%) cases, bullous pemphigoid in 7 (7.7%) cases, alopecia in 6 (6.6%) cases, disseminated maculopapular rash in 5 (5.5%) cases, delayed local reactions in 5 (5.5%) cases, herpes zoster in 4 (4.4%) cases, and exacerbation of psoriasis in 4 (4.4%) cases. (b) Number of vaccine doses received prior to the onset of adverse events in 91 cases: one dose in 35 (38.5%) cases, two doses in 21 (23.1%) cases, three doses in 21 (23.1%) cases, four doses in 5 (5.5%) cases, five doses in 8 (8.8%) cases, and unknown in 1 (1.1%) case. (c) Booster vaccination status after the development of adverse events in 91 cases: received in 22 (24.2%) cases, not received in 44 (48.4%) cases, and unknown in 25 (27.5%) cases. (d) Treatments for cutaneous adverse events in 91 cases: topical corticosteroids in 63 (69.2%) cases, oral antihistamines in 45 (49.5%) cases, oral corticosteroids in 46 (50.5%) cases, oral cyclosporine in 1 (1.1%) case, steroid pulse therapy in 3 (3.3%) cases, high-dose intravenous immunoglobulin therapy in 1 (1.1%) case, anti-IL-4/13 receptor antibody administration in 1 (1.1%) case, topical JAK inhibitor in 1 (1.1%) case, phototherapy in 8 (8.8%) cases, other treatments in 6 (6.6%) cases, and no treatment in 12 (13.2%) cases.

The main cutaneous adverse events were EM (19 (20.9%)), BP (7 (7.7%)), and alopecia (6 (6.6%)). The cutaneous adverse events categorized as “Others” included purpura simplex, Stevens-Johnson syndrome, pemphigus, peripheral vascular disease, pustular psoriasis, lymph edema, mixed connective tissue disease, exacerbation of prurigo, adult Still's disease, sarcoidosis/exacerbation of sarcoidosis, polymorphic chronic prurigo, prurigo nodularis, generalized morphea, localized scleroderma, drug eruption/eczema, acute generalized exanthematous pustulosis, granular C3 dermatosis, cellulitis, exacerbation of dermatomyositis, and erythroderma (Figure 2a).

The number of vaccine doses received before the onset of adverse events was 1 dose (35 (38.5%)), 2 doses (21 (23.1%)), 3 doses (21 (23.1%)), 4 doses (5 (5.5%)), 5 doses (8 (8.8%)), and unknown (1 (1.1%)) (Figure 2b). A total of 22 (24.2%) underwent booster vaccination even after the manifestation of adverse events, whereas 44 (48.4%) did not receive a booster dose subsequent to the development of side effects (Figure 2c). Among those 22 (24.2%) who received a booster dose, 7 (7.7%) developed similar symptoms.

Treatments for the cutaneous adverse events included topical corticosteroids (63 (69.2%)), oral antihistamines (45 (49.5%)), oral corticosteroids (46 (50.5%)), oral cyclosporine (1 (1.1%)), steroid pulse therapy (3 (3.3%)), high-dose intravenous immunoglobulin therapy (1 (1.1%)), anti-IL-4/13 receptor antibody administration (1 (1.1%)), topical JAK inhibitor (1 (1.1%)), phototherapy (8 (8.8%)), other treatments (8 (8.8%)), and no treatment (12 (13.2%)) (Figure 2d). Other treatments included intravenous glycyrrhizin therapy (1 (1.1%)), topical prostaglandin ointment (1 (1.1%)), oral etretinate (1 (1.1%)), oral anhydrous chlorpheniramine (1 (1.1%)), intravenous acyclovir therapy (2 (2.2%)), and oral amenamevir (2 (2.2%)).

Characteristics of major cutaneous adverse events

In the detailed survey, we further analyzed the characteristics of the adverse events for which there were the top three reported cases.

EM was observed in 19 (20.9%) out of 91 cases. The mean age was 62.5 years (range: 19-81 years), and the male-to-female ratio was 4:15. The patient background (N=19) included concomitant urticaria in 1 (5.3%) and malignancy in 2 (10.5%). The mean latency from the last vaccination to EM onset was 4.5 days (range: 1-20 days), and the mean duration from onset to resolution was 34.2 days (range: 1 day to 1 year).

**Figure 3:**
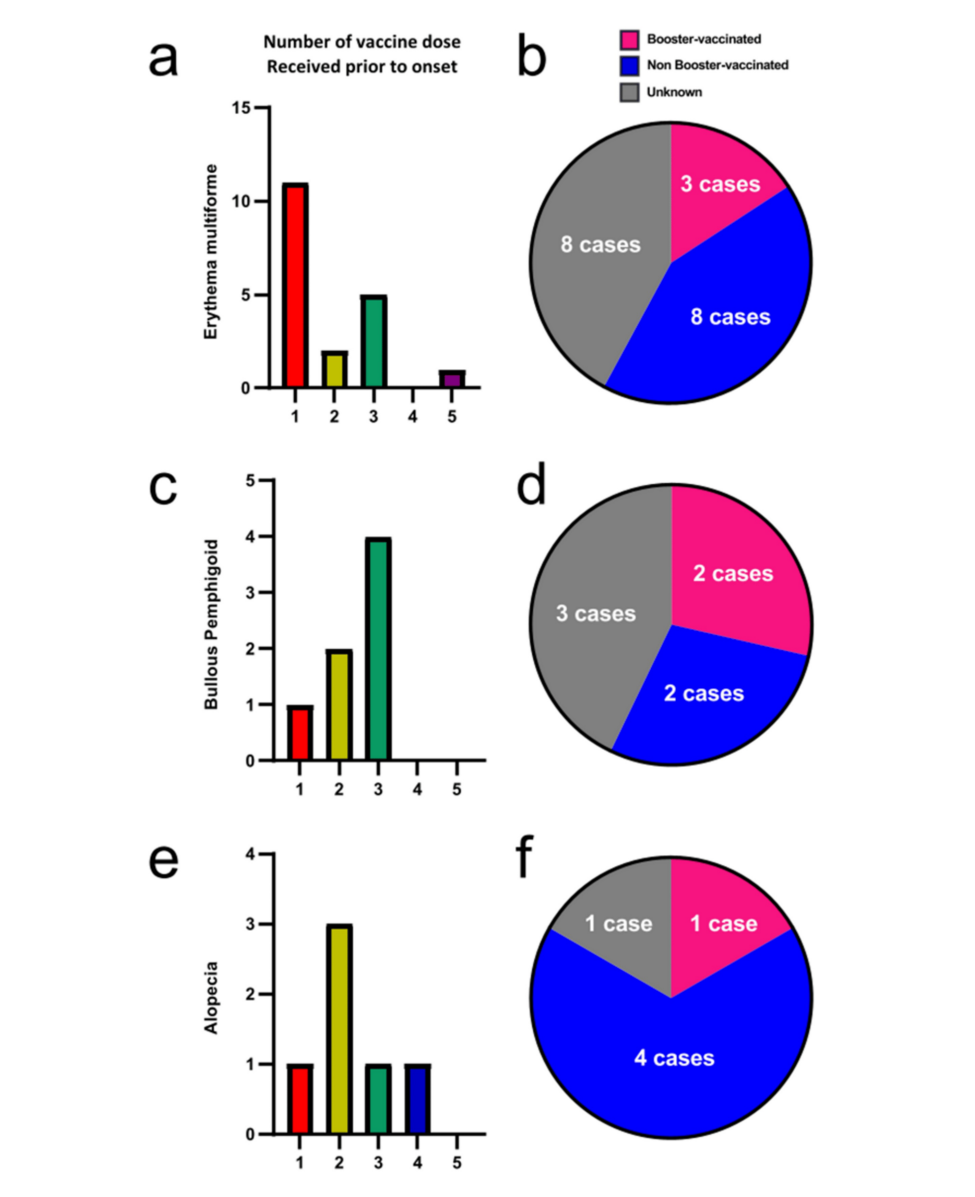
Characteristics of common cutaneous adverse events in the detailed survey (a) Number of vaccine doses received prior to the onset of adverse events in cases of EM, (c) BP, and (e) alopecia. (b) Booster vaccination status after the development of cutaneous adverse events in cases of EM, (d) BP, and (f) alopecia. A total of 19 cases of EM, 7 cases of BP, and 6 cases of alopecia were observed in the detailed survey.

Initial treatments included topical corticosteroids in 15 (78.9%), oral antihistamines in 13 (68.4%), and oral corticosteroids in 11 (57.9%). The number of vaccine doses before EM onset was 1 in 11 (57.9%), 2 in 2 (10.5%), 3 in 5 (26.3%), and 5 in 1 (5.3%) (Figure 3a). Three (15.8%) cases were re-vaccinated after developing EM (Figure 3b). One case developed EM after both Pfizer-BioNTech (BNT162b2) and delayed local reactions (mRNA-1273) COVID-19 vaccines.

BP was observed in 7 (7.7%) out of 91 cases. The mean age was 75.3 years (range: 64-88 years), and the male-to-female ratio was 5:2. The patient's background (N=7) included psoriasis vulgaris in 1 (14.3%), concomitant malignancy in 2 (28.6%), and diabetes mellitus in 3 (42.9%) (including 2 cases taking dipeptidyl peptidase 4 (DPP-4) inhibitors). The mean latency from the last vaccination to BP onset was 36.8 days (range: 1-112 days), and the mean duration from onset to resolution was 150.4 days (including ongoing cases).

Initial treatments included topical corticosteroids in 6 (85.7%), oral antihistamines in 3 (42.9%), and oral corticosteroids in 7 (100%). The number of vaccine doses before BP onset was 1 in 1 (14.3%), 2 in 2 (28.6%), and 3 in 4 (57.1%) (Figure 3c). Two (28.6%) cases were re-vaccinated after developing BP (Figure 3d).

Alopecia was observed in 6 (6.6%) out of 91 cases, all of which were female. The mean age was 63.2 years (range: 51-79 years). No notable patient background was observed (N=6). The mean latency from the last vaccination to alopecia onset was 10.8 days (range: 3-15 days), and the mean duration from onset to resolution was 300 days (including ongoing cases).

Initial treatments included topical corticosteroids in 6 (100%), oral antihistamines in 1 (16.7%), oral corticosteroids in 1 (16.7%), and ultraviolet therapy in 1 (16.7%). The number of vaccine doses before alopecia onset was 1 in 1 (16.7%), 2 in 3 (50.0%), 3 in 1 (16.7%), and 4 in 1 (16.7%) (Figure 3e). One (16.7%) case was re-vaccinated after developing alopecia (Figure 3f).

Thus, we have described the characteristics of the adverse events that were frequently reported in the detailed survey. However, it is important to note that this study is retrospective in nature, and as such, it cannot definitively prove a direct causal relationship between vaccine administration and the reported adverse events.

## Discussion

This initial nationwide survey revealed the clinical spectrum of cutaneous adverse events following COVID-19 vaccination in Japan. In the initial questionnaire survey, the most frequently encountered cutaneous events were urticaria (49 (74.2%)), delayed local reactions (37 (56.1%)), EM (31 (47.0%)), and alopecia (30 (45.5%)), consistent with previous reports from other countries [6,17-21]. The detailed survey of 91 cases provided further insights into the characteristics of cutaneous adverse events. These detailed survey results were affected by the bias in the institutions that responded to the initial questionnaire. EM was the most common specific event (19 (20.9%)), followed by BP (7 (7.7%)), and alopecia (6 (6.6%)).

The mean latency from the last vaccination to the onset of cutaneous adverse events was 13.1 days, with most events occurring within 2 weeks after vaccination. This is consistent with previous reports and suggests that close follow-up during this period may be important for the early detection and management of cutaneous adverse events [18,19]. 

The role of booster doses in cutaneous adverse events remains unclear. While many cases develop symptoms after the primary series, cutaneous adverse events are also observed for the first time after the second or third dose, including booster vaccinations. In this study, among the three most frequently reported conditions, EM is more frequently observed after the first dose, whereas BP and alopecia are more commonly reported in patients who have received multiple doses, including boosters. However, no definitive conclusions can be drawn regarding the relationship between vaccine dose number and disease onset.

Some patients develop cutaneous adverse events for the first time after multiple vaccine doses while others do not experience recurrence even after revaccination. Given this variability, continuous follow-up is essential to ensure appropriate risk assessment and patient management.

Concerning the treatment, the majority of cutaneous events were self-limited or responded well to treatment with topical corticosteroids, oral antihistamines, or oral corticosteroids. However, some cases required more intensive therapies such as steroid pulse therapy, high-dose intravenous immunoglobulin therapy, or biologic agents. Dermatologists should be prepared to provide appropriate care for patients who develop severe or refractory cutaneous adverse events following COVID-19 vaccination.

Furthermore, EM is one of the cutaneous adverse events to COVID-19 vaccines [22], but it is considered relatively rare [23]. However, in this detailed survey, it was the most frequently observed event, with 19 (20.9%) out of 91 cases. The male-to-female ratio of 4:15, with a higher incidence in females, is consistent with previous reports [23]. Several studies have suggested that women generally exhibit a higher antibody response to vaccination compared to men and experience more adverse events post-vaccination [24-28]. Considering that EM is a complication that also occurs with several other vaccines, such as mumps, measles, and hepatitis B [22], it may be a relatively common complication, as suggested by the results of this survey. In cases of EM, there is a higher risk of developing more serious adverse events such as EM major, Stevens-Johnson syndrome, or toxic epidermal necrolysis [23]. Therefore, it is important to closely monitor patients during the next vaccination. However, considering that viral infections themselves can also trigger the onset [29,30], re-vaccination is not considered a contraindication.

BP has been reported as a rare cutaneous adverse event following COVID-19 vaccination [16,17]. However, in the present detailed survey, BP was observed at a high rate of 7 (7.7%) out of 91 cases, which may be attributed to the presence of comorbidities. Among the 7 cases that developed BP, 2 (28.6%) had malignant tumors, and 2 (28.6%) were taking DPP-4 inhibitors, a class of medications used for the treatment of diabetes mellitus. Although the causal relationship between malignant tumors and BP has not been clearly established, a higher prevalence of malignant tumors has been reported in BP patients compared to the general population [31-34], and the association has been suggested. Moreover, a significant association has been found between the use of DPP-4 inhibitors, a diabetes medication, and the development of BP [35]. BP has also been reported to be associated with various drugs such as antihypertensive drugs, diuretics, and antibiotics [36,37]. Furthermore, among the 6 (6.6%) cases with malignancy in the entire cohort, 5 (83.3%) were male and 1 (16.7%) was female. Similarly, among the 4 (4.4%) cases with diabetes mellitus, 3 (75.0%) were male (including 2 (50.0%) who were taking DPP-4 inhibitors) and 1 (25.0%) was female. Given that these underlying conditions are known risk factors for BP, the higher prevalence of BP in men in this study may be partially explained by the greater proportion of male patients with malignancy or diabetes mellitus. For the early detection and treatment of vaccine-induced BP, it is important to inquire about the presence of malignant tumors and the use of high-risk medications. Additionally, since there have been reports of a high risk of worsening or reactivation with the next vaccine dose after the initial onset [16], close monitoring during the next vaccination is crucial.

COVID-19 vaccines can cause various types of alopecia, including telogen effluvium and alopecia areata [38-40]. They have also been reported to induce severe forms of alopecia areata such as alopecia totalis and alopecia universalis [41,42]. In this survey, 6 (6.6%) out of 91 cases developed alopecia, suggesting that the association between vaccines and alopecia cannot be denied. On the other hand, alopecia has been associated with atopic predisposition [43,44] and psychological stress [45], and it is thought that the psychological stress caused by the COVID-19 pandemic may trigger or exacerbate alopecia [46]. COVID-19 vaccination is recommended for all patients with alopecia areata who do not have known allergies to vaccine components [39]. However, alopecia is a condition that affects appearance, and the average duration from onset to recovery is 300 days (including cases still under treatment), which means that it is an adverse event that greatly impairs patients' quality of life. It was found that patients tend to avoid re-vaccination (4 (66.7%) out of 6 cases, with 1 (16.7%) case unknown, did not receive re-vaccination).

Although the present study provides valuable insights, it is important to recognize its several limitations. First, the response rate to the initial questionnaire survey was relatively low (66 facilities (52.4%)), which might have introduced a selection bias. As the characteristics of non-responding facilities are unknown, it is unclear whether their patient populations or diagnostic criteria differed from those of the participating institutions. Second, the detailed survey was based on a small sample size of 91 cases, which may not fully represent the entire spectrum of cutaneous adverse events. Additionally, institutions that responded to the survey may have been more likely to report severe or unusual cases, potentially leading to an overrepresentation of such events. Conversely, mild cases might have been underreported, as they may not have been considered significant enough for submission. Furthermore, the differences in disease profiles between the initial and detailed surveys were likely influenced by the variation in the number of participating institutions. These factors highlight the need for a cautious interpretation of the results, as selection bias may have affected the overall incidence and distribution of cutaneous adverse events in this study. The initial survey encompassed a broad investigation targeting 126 major dermatological institutions. However, the detailed survey was limited to 15 facilities that voluntarily agreed to participate, and the case registration methods varied among facilities. Furthermore, there is a possibility that severe or rare cases were more likely to be reported due to their memorable nature. These differences in disease profiles did not arise from the intentional selection of participating institutions but rather emerged naturally from the institutions that chose to report cases. The differences observed in the disease profiles between the initial survey and the detailed survey are likely influenced by the varying levels of cooperation from participating institutions. The initial survey involved a broad inquiry to 126 major dermatology institutions, leading to reports of relatively mild cutaneous conditions. However, the detailed survey saw greater participation from institutions that manage more severe cases, which resulted in a focus on more serious conditions. This discrepancy in disease profiles was not due to intentional selection of the participating institutions but, rather, arose naturally from the institutions that chose to report their cases. Therefore, the study design itself does not introduce bias, though the variation in case severity between institutions contributed to differences in the reported profiles. Third, the causality between COVID-19 vaccination and the reported cutaneous events cannot be definitively established due to the retrospective nature of the study. Further large-scale prospective studies are needed to confirm our findings.

Despite these limitations, this study offers valuable information on the clinical spectrum and characteristics of cutaneous adverse events following COVID-19 vaccination in Japan. Dermatologists should be aware of the potential cutaneous adverse events and provide appropriate care for affected patients. Continued monitoring and reporting of cutaneous adverse events will be essential to ensure the safety and public confidence in COVID-19 vaccines.

## Conclusions

This nationwide survey revealed that, though rare, cutaneous adverse events following COVID-19 vaccination occur frequently enough to warrant presentation in dermatology clinics in Japan and could influence dermatological practice. In the detailed survey, the most commonly reported events were EM, urticaria, BP, and alopecia. Although individuals with pre-existing skin diseases appeared to develop cutaneous adverse events in some cases, the study design does not allow for a definitive risk assessment.

While most events were self-limited or manageable with treatment, dermatologists should be aware of the potential for severe or refractory cases and consider individualized patient management. Continuous monitoring and further research on cutaneous adverse events following vaccination may be important to better understand their long-term impact and implications for public health.

## Appendices

Appendix A 

**Table 2:** Questionnaire for the initial survey This table presents the questionnaire items used in the initial nationwide survey, which was sent to 126 dermatology facilities. Respondents were asked to select all cutaneous adverse events following COVID-19 vaccination that were experienced at their institution (multiple answers allowed). If none of the listed options applied, participants were instructed to specify any other events under “Others.”

Question	Response Options	Example
Please select all cutaneous adverse events following COVID-19 vaccination that your institution has experienced (multiple answers allowed).	Urticaria	NA
	Angioedema	NA
	Anaphylaxis	NA
	Delayed local reactions	NA
	Erythema multiforme	NA
	Disseminated maculopapular rash	NA
	Lupus erythematosus	NA
	IgA vasculitis	NA
	Leukocytoclastic vasculitis	NA
	Other vasculitis	NA
	Pityriasis rosea	NA
	Chilblains-like eruption	NA
	Herpes zoster	NA
	Alopecia	NA
	Vitiligo	NA
	Exacerbation of atopic dermatitis	NA
	Others (please specify)	NA

Appendix B 

**Table 3:** Questionnaire for the detailed survey This table outlines the questionnaire items used in the detailed survey, which collected more comprehensive information on all cases of cutaneous adverse events following COVID-19 vaccination. Each facility was asked to provide patient demographics, vaccination details, latency periods, treatment approaches, and outcomes.

Question No.	Question	Response Type / Options	Example
1	Patient age (years)	Numeric (e.g., 18–95)	NA
2	Patient sex	Male / Female / Other	NA
3	Pre-existing medical conditions	Atopic dermatitis / Psoriasis vulgaris / Urticaria / Malignancy / etc.	NA
4	Latency from the last vaccination to onset (days)	Numeric	NA
5	Duration from onset to resolution (days)	Numeric / Ongoing	NA
6	Number of vaccine doses received before onset	1 / 2 / 3 / 4 / 5 / Unknown	NA
7	Booster vaccination after adverse event	Yes / No / Unknown	NA
8	Outcome after booster (if applicable)	Similar symptoms / No similar symptoms	NA
9	Treatment(s) for the cutaneous adverse event	Topical corticosteroids / Oral antihistamines / Oral corticosteroids / Oral cyclosporine / etc.	NA
10	Other treatments (if any)	Free text	NA
11	Additional notes (if needed)	Free text	NA
